# Enhancing Modulation of Thermal Conduction in Vanadium Dioxide Thin Film by Nanostructured Nanogaps

**DOI:** 10.1038/s41598-017-07466-4

**Published:** 2017-08-02

**Authors:** Hwan Sung Choe, Joonki Suh, Changhyun Ko, Kaichen Dong, Sangwook Lee, Joonsuk Park, Yeonbae Lee, Kevin Wang, Junqiao Wu

**Affiliations:** 1 Department of Materials Science and Engineering, University of California, Berkeley, Berkeley, CA 94720 USA; 2 Department of Precision Instrument, Tsinghua University, Beijing, 100084 China; 3 Department of Materials Science and Engineering, Stanford University, Stanford, CA 94305 USA; 4 Materials Sciences Division, Lawrence Berkeley National Laboratory, Berkeley, CA 94720 USA

## Abstract

Efficient thermal management at the nanoscale is important for reducing energy consumption and dissipation in electronic devices, lab-on-a-chip platforms and energy harvest/conversion systems. For many of these applications, it is much desired to have a solid-state structure that reversibly switches thermal conduction with high ON/OFF ratios and at high speed. Here we describe design and implementation of a novel, all-solid-state thermal switching device by nanostructured phase transformation, i.e., modulation of contact pressure and area between two poly-silicon surfaces activated by microstructural change of a vanadium dioxide (VO_2_) thin film. Our solid-state devices demonstrate large and reversible alteration of cross-plane thermal conductance as a function of temperature, achieving a conductance ratio of at least 2.5. Our new approach using nanostructured phase transformation provides new opportunities for applications that require advanced temperature and heat regulations.

## Introduction

Control of heat conduction across interfaces would open up tremendous opportunities in advanced thermal energy management, to achieve super-stable temperature control, rapid temperature cycling, or pulsed thermal power operation^1–7^. In this regard, thermal switches, which alter thermal conductance between ON and OFF states, have attracted considerable attention. In current technologies based on microfabrication, thermal switches are mainly achieved by connecting and breaking two surfaces with the motion of liquid droplets^8–10^ or solid membranes actuated with electric field^11^ or piezoelectric motors^12^. However, these non-monolithic approaches with moving parts often suffer from one or more of the following limitations: materials instability and toxicity, scalability, poor integrability with other devices, low ON/OFF ratio, low speed, and high energy consumption.

**Figure 1:**
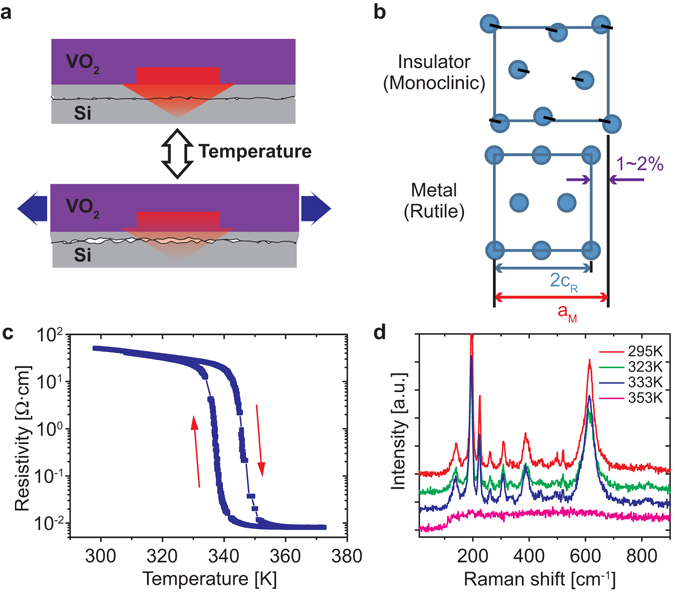
Concept of the nanostructured phase transformation and as-grown VO_2_ film properties. (**a**) Schematic of the nanostructured phase transformation with a nanogap, showing contact pressure and area modulated by the phase transition strain. (**b**) Illustration of the lattice structure change of VO_2_ across the phase transition between the insulating and metallic phases. In the schematics, the cyan circle indicates vanadium atom, and oxygen atoms are omitted for clarity. (**c**) Semi-log plot of resistivity of the VO_2_ film as a function of temperature during the heating and cooling process. (**d**) Raman spectra of the VO_2_ film at different temperatures showing the phase transition between 333 and 353 K.

Solid-state phase transition materials such as vanadium dioxide (VO_2_)^13, 14^, germanium antimony tellurium (GeSbTe) ternary alloys^15^, and lead zirconium titanate (PZT)^16^ are among the most attractive candidates for high-performance thermal switches: high speed of the phase transition^17, 18^, intrinsic difference in thermal conductivities of each phase^13, 15, 16^, and reversible phase transitions activated by diverse external stimuli such as electric^16, 19, 20^, optical^21–23^, mechanical^24, 25^, or thermal^13, 15^ ones. Demonstrated performance of the thermal switching based on these materials is, however, severely limited in one or more aspects of switching speed, ON/OFF ratio, and/or energy efficiency. In this context, we develop a thermal switching structure (TSS) operated on the basis of open/close of a 1~2 nm nanogap activated by nanostructured phase transformation in a VO_2_ thin film. The approach is based on a recently developed, intriguing, multifunctional material composite that exhibits superior performance of micro-scale actuation. The system is composed of VO_2_, which possesses a ferroelastic phase transition, coupled mechanically to a structural material. The shape and mechanical, thermal, electrical and optical properties of the composite can be drastically and reversibly modified under external stimuli. At 68 °C, VO_2_ undergoes a coupled metal-insulator and rutile-monoclinic phase transition, where the effective lattice constant changes abruptly by up to 1~2% (Fig. 1b). Owing to the large work density of the VO_2_ phase transition, these actuators can deliver simultaneously high force and large stroke of actuation, as opposed to other actuation mechanisms where only one is high. As shown in Fig. 1a, the TSS is mainly composed of a thin VO_2_ layer stacked onto a polycrystalline Si layer, with a nanogap created in the Si layer. The layers are deposited at temperatures higher than the VO_2_ phase transition temperature (*T*_*PT*_), such that at *T* > *T*_*PT*_ the nanogap interface is largely conformal. As temperature is cooled across the phase transition to *T* < *T*_*PT*_, the shape change of VO_2_ layer pulls the neighboring Si layer away from the Si layer below that, strongly reducing the contact pressure and area between these two layers. Heat conduction across the interface is thus proportionally reduced. Our TSS demonstrates dramatic enhancement of the thermal-switching ON/OFF ratio, by a factor of ~6.7, compared to VO_2_ film devices without incorporation of the nanogap.

## Results

The VO_2_ thin films were grown using pulsed laser deposition on the surface of a poly-Si layer that was prepared with low-pressure chemical vapour deposition. Electrical characterization (Fig. 1c) of the VO_2_ film showed over three orders of magnitude change in its resistivity, and Raman spectroscopy (Fig. 1d) also confirmed the phase transition from the insulating phase to the metallic phase at around 341 K, consistent with previous studies^26^.

**Figure 2:**
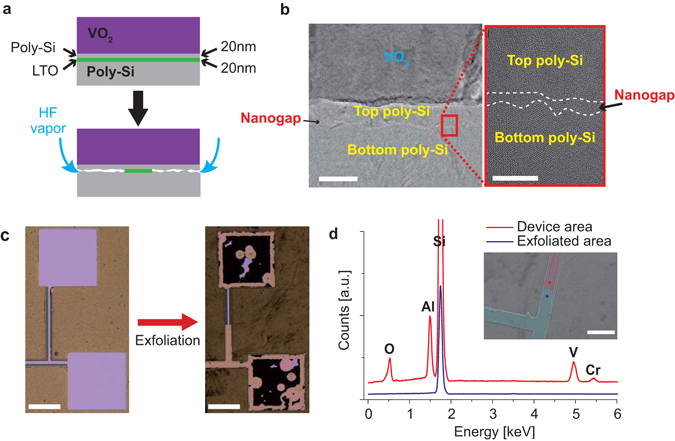
(**a**) Illustration of main process of the interface fabrication. The interface is formed by removal of the sacrificial LTO layer between the poly-Si layers with HF vapour etching. (**b**) Cross-sectional AC-HRTEM images of the interface area. The white dashed lines in the red box are drawn along the edge of the poly-Si layer. The continuum of atoms filling the nanogap were formed during the TEM sample preparation. Scale bar: 40 nm (left), and 8 nm (right). (**c**) Optical microscopic images of the electrodes of the thermal switch before (left) and after (right) tape exfoliation. Scale bar: 160 μm (both left and right). (**d**) EDX spectra of exfoliated and un-exfoliated areas of the thermal switching device showing that the exfoliation indeed peels off the VO_2_ layer. Inset shows SEM image of the areas where EDX spectra were taken. False colors are added in exfoliated and un-exfoliated areas for eye guidance. Scale bar: 80 μm.

In addition to the VO_2_ layer as the activation material, the other key component of the device is the nanogap embedded in the poly-Si layer that allows the open/close motion. This was created as illustrated schematically in Fig. 2a (see Experimental Section for details). A ~20 nm sacrificial layer of low-temperature silicon dioxide (LTO) was sandwiched by the poly-Si layers, which was selectively removed using a hydrofluoric acid (HF) vapour etch. In particular, the HF vapour was utilized to etch the LTO entering from the side opening, causing the top poly-Si layer to collapse onto the bottom poly-Si layer with good conformation. Cross-sectional aberration-corrected high-resolution transmission electron microscopy (AC-HRTEM) of the interface area (Fig. 2b) revealed 1~2 nm interface roughness from both the top and bottom poly-Si layers, which indicates successful formation of a 1~2 nm nanogap in the structure. The existence of the designed nanogap in the device structure is further verified as follows. First, the layer above the nanogap was able to be readily peeled off by mechanical exfoliation with a scotch tape. Figure 2c shows top-view optical images of the TSS before and after the mechanical exfoliation. Secondly, the exposed area, in contrast to the un-exfoliated area, shows absence of vanadium signal with energy dispersive x-ray (EDX) analysis (Fig. 2d).

Modulation of the contact area across the nanogap of the TSS is expected to switch cross-plane thermal conduction of the structure, which was measured by the differential 3ω technique at variable background temperatures^27^. For clarity, the TSSs are hereby termed as “VO_2_+NG” as they incorporate both the VO_2_ layer and the nanogap. To extract thermal conductance of the VO_2_-interface component within the multiple-layer stacked structure, we also prepared three control devices (Fig. S1): one with the same structure as the “VO_2_+NG” device but without the VO_2_ layer and the interface, hereby termed as “Si-only”. In addition, an nanogap-only device (“NG”) and a VO_2_-only device (“VO_2_”) were also prepared with otherwise identical structure under identical growth condition. According to the differential 3ω method, these different device structures in Fig. S1 can be utilized to investigate the modulation of thermal conduction across both the nanogap and the VO_2_ layer, driven by the neighboring structural phase transition, using the other structures as control devices.

As the width of the electric heater patterned for the 3ω technique (Fig. S9k) is much larger than the thickness of the entire stack, heat created in the top heater is considered to flow only vertically, resulting in a one-dimensional heat transfer problem. As a result, thermal response of a device containing any layer should be a linear combination of *ΔT*_*reference*_ and *ΔT*_*layer*_. For example, thermal resistance of the “VO_2_+NG” device would be equal to the “Si-only” device in series with the “VO_2_+NG” layer, assuming interfacial resistances negligible;^27^ consequently, effective thermal conductivity of the VO_2_+NG layer is given by

1 $${\kappa }_{V{O}_{2}+NG}=\frac{{I}_{\omega }^{2}\cdot R\cdot {t}_{V{O}_{2}+NG}}{2bl({\rm{\Delta }}{T}_{V{O}_{2}+NG}-{\rm{\Delta }}{T}_{Si-only})}$$

where ${t}_{{{VO}}_{2}+{NG}}$, ${{\rm{\Delta }}T}_{{Si}-{only}}$, ${{\rm{\Delta }}T}_{{{VO}}_{2}+{NG}}$, *2b*, and $l$ are the effective thickness of the VO_2_ and nanogap layer, temperature oscillation amplitude of the “Si-only” device, temperature oscillation amplitude of the “VO_2_+NG” device, width, and length of the aluminum (Al) heater line (Fig. 9Sk), respectively^27–29^.

**Figure 3:**
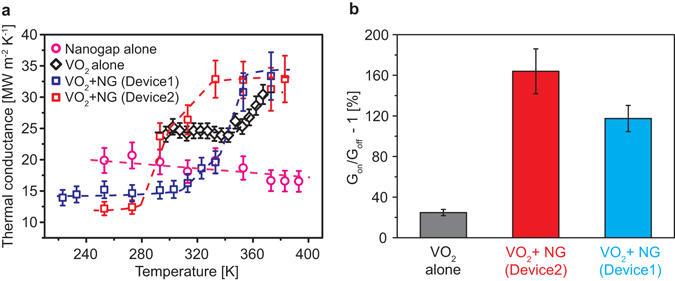
(**a**) Thermal conductance versus temperature plots of the nanogap, VO_2_ film alone, “VO_2_+NG” Device1, and “VO_2_+NG” Device2, respectively. 10% errors are estimated considering the error of thicknesses and measurements. The dashed lines are added as guides to the eye. (**b**) Comparison of thermal switching performance of VO_2_ film, “VO_2_+NG” Device2, and “VO_2_+NG” Device1 based on (**a**).

Figure 3a shows temperature dependence of the cross-plane thermal conductance of the “VO_2_+NG”, “NG” and “VO_2_” layers measured with the differential 3ω method, using the “Si-only” device as the reference. For the two “VO_2_+NG” devices, two different thicknesses of the topmost Al_2_O_3_ capping layer, being 25 nm (Device1) and 60 nm (Device2) respectively, were prepared as the electrical isolation layer between the Al heater and the underlying VO_2_ film. The phase transition temperature of the VO_2_ film in Device2 is lowered from the natural temperature (68 °C) to near room temperature as shown in Fig. S2. This is possibly attributed to the geometrical confinement effects by the upper Al_2_O_3_ capping layer and the lower poly-Si layer^30^. We analyze the behavior of each device as follows.

First of all, the VO_2_ layer shows ~26% increase in thermal conductance as the VO_2_ switches from the insulating to the metallic phase, resulting in a G_on_/G_off_ of ~1.26 for the VO_2_ layer. From the thickness of the VO_2_ layer, the total thermal conductivity of the VO_2_ layer is estimated to increase by ~0.76 W m^−1^ K^−1^ when going from the insulating to the metallic phase. Hall effect measurement reveals that the electrical conductivity of the metallic phase of the VO_2_ layer is ~740 Ω^−1^ cm^−1^. From the Wiedemann-Franz law with the Sommerfeld value of the Lorenz number, the electronic contribution to the thermal conductivity of the VO_2_ is estimated to be ~0.67 W m^−1^ K^−1^ (Fig. S3), which is generally consistent with the increase in the total thermal conductance of the VO_2_ layer. This is in good agreement with previous work that shows the validity of the Wiedemann-Franz law in the metallic state of polycrystalline VO_2_ thin films^13^. Secondly, assuming that the effective average thickness of the nanogap is ~2 nm, supported by AC-HRTEM analysis (Fig. 2b), its thermal conductivity is determined from the “NG” device to be ~0.02 W m^−1^ K^−1^, and is generally a constant as temperature varies, as shown in Fig. S3.

Most importantly, extracted from 3ω signals (Fig. S4), the “VO_2_+NG” devices exhibit a remarkable enhancement of the cross-plane thermal switching compared to the “VO_2_” devices. The ON/OFF switching ratio of the thermal conductance (G_on_/G_off_) of the “VO_2_”, “VO_2_+NG” Device1 and “VO_2_+NG” Device2 is 1.26, 2.39 and 2.75, respectively, corresponding to an enhancement of up to a factor of ~ 6.7 (26% increase in thermal conductance in the “VO_2_” device is improved to 175% in the “VO_2_+NG” Device) when the nanogap is inserted (Fig. 3b). This result indicates that insertion of the nanogap into the poly-Si buffer layer significantly improves the thermal switching performance by utilizing the VO_2_ phase transition strain to modulate the contact area and, consequently, thermal conductance. Therefore, the mechanism of thermal conductance switching in the “VO_2_+NG” devices is different from that in the “VO_2_” device, where the ON-state is more thermally conductive than the OFF-state owing solely to free electrons released in the metallic phase.

**Figure 4:**
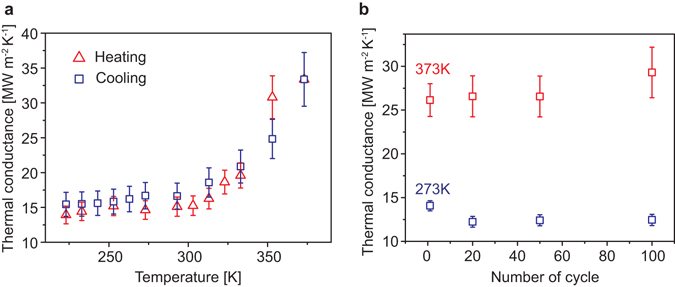
(**a**) Thermal conductance versus temperature plot of “VO_2_+NG” Device1 during heating and cooling. (**b**) Thermal conductance switching of the “VO_2_+NG” Device1 from (**a**) during sequential cooling and heating between 373 K and 273 K, corresponding to alternation between the metal and insulator phases of VO_2_. The ramp rate of temperature was 5 K min^−1^ for both heating and cooling.

The thermal switching of the “VO_2_+NG” nanomechanical devices is reversible and stable. Figure 4a shows thermal conductance of the Device1 during increase and decrease of background temperature. The conductance during the heating agrees well with that in the cooling with a small hysteresis. The hysteresis is narrower than that of the electrical conductivity in Fig. 1c, possibly due to the slow temperature variation in the former: the device was stabilized for 45 minutes prior to measurements of the thermal conductance at each temperature point. The nanostructuring and patterning for device fabrications may also introduce various defects in the VO_2_ layer, which can act as additional nucleation sites for the transition, effectively reducing the hysteresis. Plotted in Fig. 4b is the repeated cycling of switching of the Device2, showing alteration of the thermal conductance between 12–14 MW m^−2^ K^−1^ for the insulating phase at 273 K and 26–28 MW m^−2^ K^−1^ for the metallic phase of VO_2_ at 373 K. The device switches with good endurance and stability up to 100 cycles without any detectable degradation in performance.

Furthermore, to investigate the switching speed of our TSS, the phase transitions of the VO_2_ layer in a “VO_2_+NG” Device1 were monitored in a cryostat by a Raman spectroscope with temperature control. It is worthy to note that phase transitions of the VO_2_ layer are responsible for the thermal switching of the “VO_2_+NG” Device1. Its Raman spectra as heating and cooling (Fig. S5) showed clear VO_2_ phase switching in <~10 s and <~30 s during 30 K heating and cooling, respectively, and demonstrated phase changes in ~3 s during 10 K heating. The measured switching speed is significantly limited by, not an intrinsic device property, but ramp rates of background temperature during heating and cooling, as well as an acquisition time to obtain the Raman spectra. To evaluate the maximum thermal switching speed, we simulated VO_2_ phase switching in our TSS as applying a stepwise heat input by Joule heating on its top (see Methods for details). The results shown in Fig. S6 revealed that the maximum switching speed can be achieved up to ~1 ms. This switching speed is much slower than the intrinsic speed of both the metal-insulator transition (MIT) and the structural phase transition in VO_2_^17, 18^, as the speed is limited by heat generation and dissipation in the structure. However, this speed is already comparable or faster than all reported solid thermal switches (see Table S1), and much faster than thermal switches involving liquids^8–10, 12^.

## Discussion

The different thermal conduction of the “VO_2_” and the “VO_2_+NG” devices implies that the inserted nanogap plays an active role in the thermal switching. To directly probe that, we performed *in-situ* SEM of the cross section of a “VO_2_+NG” device under temperature variation (Fig. S7). The nanogap is not directly resolved because of limitation in the SEM resolution. However, the thickness of the poly-Si layer above the nanogap varies reversibly with temperature cycling across the phase transition of VO_2_ (Fig. S7b,c). It is clear that the transformation strain of the VO_2_ film imposes stress onto the neighboring top poly-Si layer which is separated by the nanogap from the bottom poly-Si layer, hence modulating the nanogap size.

To further understand the thermal switching mechanism of the device, we simulated the heat conduction and radiation across the nanogap by analytical calculations. The total heat transfer coefficient attributed to radiative and conductive heat transfer across the nanogap between the poly-Si layers was first calculated using the Polder and Van Hove model^31, 32^. The calculation using the poly-Si permittivity^33^ show radiative heat transfer coefficient carried by far-field waves or evanescent waves (Fig. S8a,b). Not surprisingly, the results at both temperatures (where the VO_2_ is in two different phases) reveal that radiative heat transfer grows exponentially as the nanogap is reduced from 10 to below 0.1 nm. Due to 1~2 nm roughness of the poly-Si surface (Fig. 2b) and hence the non-uniform thickness of the nanogap at nanoscale, we calculated the total heat transfer coefficient across a simplified gap structure, based on the results in Fig. S8a,b: a Si pyramid approaching a plane of Si (inset of Fig. S8c). We assume an initial gap size of 2 nm, and that the heat conduction under direct contact is equal to the amount of radiative heat transfer at 1 Å gap^34^. Figure S8c,d show the calculated heat transfer coefficients of radiation and conduction across the structure as the gap is closed when the pyramid approaches the other plane. The result shows that the total heat transfer is by radiation at 2 nm gap at 253 K (OFF state of the thermal switch), and the conduction component of heat transfer increases as the gap is reduced. The experimental result of G_on_/G_off_ ~ 2.75 from our TSS (red block in Fig. 3b) can be reproduced if the nanogap shrinks from 2 nm in the OFF state to ~1.5 nm in the ON state. We note that, as both the direct heat conduction and near-field heat radiation contribute to the total heat transfer, the thermal conduction would not be zero even when the nanogap is completely open. This limits the maximum G_on_/G_off_ from divergence. In the ideal scenario assuming that the nanogap can vary from ~1 μm (fully open) to a fully conform contact, the calculation shows that G_on_/G_off_ reaches up to two orders of magnitude. Overall, both the *in-situ* SEM imaging and the simulation support the active role of the nanogap in the heat transfer modulation.

We also estimated the energy density required for our TSS. Using the known latent heat^35, 36^ of MIT in VO_2_, the consumed energy density is ~47 pJ μm^−2^ for a 200 nm VO_2_ layer used. Assuming the same active layer thickness (200 nm for all devices), this value is about 1/5 of that in thermal switches based on the GeSbTe phase change materials system^15^, but a factor of 40 higher than that based on the PZT ferroelectric system^16^. Compared to the PZT devices, the merit of our device therefore lies in its higher ON/OFF ratio, as shown in Table S1, with sacrifice in higher energy consumption.

In summary, we demonstrated a thermal switching in a solid-state nanomechanical thin-film structure based on nanostructured phase transformation. A high ON/OFF ratio of ~2.75 in cross-plane thermal conductance is achieved in the device as the contact area across a nanogap is modulated by the phase transition strain of the VO_2_ layer. This is a 670% enhancement from the thermal switching based on the metal-insulator transition of VO_2_ alone. VO_2_ is non-toxic, and thermally and chemically stable (VO_2_ micro-actuators show no degradation after millions of operation)^37^, its phase transition in a TSS is with high speed (intrinsically ~picosec, leading to device speed ~1 kHz limited purely by thermal dissipation)^38^, and the transition temperature can be shifted to other temperatures with chemical doping^19^. The fact that the phase transition can be driven thermally^13^, optically^21^, electrically^19^ and electrostatically^20^ promises versatility and flexibility in operation and performance of the thermal switch in nanoelectromechanical systems. The demonstrated solid-state thermal switching is expected to open new opportunities for applications that require advanced temperature and heat regulations.

## Methods

### VO_2_ thin films growth and characterization

All VO_2_ thin films used in this study were grown on either lightly p-doped (20–30 Ω·cm) single crystalline (100) silicon substrate or undoped polycrystalline silicon thin films by pulsed laser deposition (PLD). A Krypton fluoride excimer laser (248 nm wavelength) was focused on a VO_2_ target (pressed 99.9% pure, powder) with a pulse repetition rate of 5 Hz and a fluence of 350 mJ cm^−2^. The deposition was done at 500 °C in 10 mTorr with 2 sccm oxygen gas flow. After finishing the deposition, the sample was cooled down to room temperature at a rate of 10 °C min^−1^ with retaining oxygen gas pressure. The thickness, crystal orientation, resistance, and phase of the as-grown film were characterized by SEM, XRD, electrical transport measurement, and Raman spectroscopy, respectively. For the resistance measurement, two probes made contacts at the opposite sides of the film grown on undoped poly-Si film, and the film resistance was recorded at variable substrate temperature. The results are used for the resistivity calculation of the VO_2_ film (Fig. 1c) with the information of its dimension.

### Device fabrication and AC-HRTEM characterization

The high-quality, undoped poly-Si and low temperature undoped silicon oxide (LTO) were deposited by low-pressure chemical vapour deposition at 615 °C and 450 °C, respectively, using reactive silane and oxygen gases in 300 mTorr (Fig. S9a). The 120 or 200 nm VO_2_ film was deposited by PLD on underlying 120 nm poly-Si/20 nm LTO/20 nm poly-Si thin-film stack (Fig. S9b), and then 70 nm Al_2_O_3_ using atomic layer deposition (ALD) (vacuum, 200 °C, trimethylaluminum and DI water as precursors) and Cr(1 nm)/Au(70 nm) layer by electron-beam evaporation to protect underlying layers during further etching processes. (Fig. S9c). The interface forms from HF vapour etch (uEtch, SPTS Technologies) (Fig. S9e), following wet chemical etch with BHF and selective RIE (100 W, 13 sccm SF_6_ and 21 sccm He) to remove the Al_2_O_3_ and poly-Si/VO_2_ layers, respectively (Fig. S9d), to allow the etchant to access the LTO layer. The Cr/Au protection layers were etched out using CR-7 chromium and TFA gold etchant, respectively (Fig. S9f). Right after the etching process, Si_3_N_4_ (10 nm) and Al_2_O_3_ (60 or 25 nm) were deposited by plasma-enhanced chemical vapour deposition (20 W, 0.9 Torr, 200 °C, 30 sccm NH_3_ and 100 sccm 10% SiH_4_ in Ar) and ALD, respectively (Fig. S9g). In particular, the thin Si_3_N_4_ layer was for prevention of the Al_2_O_3_ deposition into the localized nanogaps at the interface during post-ALD process, and the additional Al_2_O_3_ layer was for electrical isolation between the VO_2_ film and the electrodes. Finally, the electrode pattern of Cr (1 nm)/Al (100 nm) for 3ω method was metallized by standard photolithography and electron beam evaporation (Fig. S9h). The yield of the TSS demonstrating proper thermal switching operation was 75% (3 out of 4 devices).

The samples for cross-sectional AC-HRTEM (Titan, FEI) analysis were prepared by focused ion beam (Helio NanoLab, FEI) and lift-out technique. All HRTEM images (Fig. 2b and Fig. S9j) were taken at 300 kV with spherical aberration correction. For high contrast of the interface, >40 nm defocus and an objective aperture were used.

### Sample preparation and experimental setup for the 3ω method

All devices were mounted on a 24-pin chip carrier with a class-A platinum thin-film resistance temperature detector (RTD) by conductive silver paint. It is worthy to note that the RTD was employed to accurately read the temperature right on the devices. Al wire bonding and silver conductive epoxy (EPO-TEK® H20E) were used for stable electrical connections between the chip carrier and the Al heater over a wide range of temperatures. The chip carrier with devices was loaded into a cryostat (CCS-400H/204, Janis) which was connected to a vacuum turbopump (T-Station 75, Edwards), a temperature controller (Model 331, Lakeshore) and a home-made BNC box. For 3ω voltage measurement of the devices, the cryostat was evacuated to ~1 × 10^−7^ Torr, and a lock-in amplifier (SR830, Stanford Research Systems), V to I converters, a multiplying DAC, and a high precision resistors (100 Ω, ± 0.005% resistance tolerance, ± 0.05 ppm °C^−1^ temperature coefficient) were wired as shown in Fig. S10. We have used a commercial fused silica substrate to benchmark our 3ω-system, and found a thermal conductivity of 1.22 W m^−1^ K^−1^ (Fig. S11) very close to the known value. Before starting to record data, the global temperature was raised up to 423 K and stayed there for ~30 min to anneal the devices. For the temperature coefficient of resistance (TCR) of each Al heater at the target temperature T_0_, I-V curves were collected at five different temperatures, T_0_-4, T_0_-2, T_0_, T_0_+2, and T_0_+4 K after temperature stabilization for 30 minutes at each temperature. The resistances of the heater at the five temperatures were obtained by a linear fitting to the I-V curve. Consequently, The TCR at T_0_ was determined by a linear-fitting. The 3ω data acquisition at T_0_ was carried out after temperature stabilization for 45 minutes, too. It is worth noting that the nanogap modulation occurs prior to the 3ω measurements, such that during the measurements, the contact area is not actuating, and the measurements in Figs 3 and 4 are static/equilibrium instead of dynamic.

### Experimental setup and condition of the Raman spectroscopic characterization for dynamic switching measurement

All devices characterized by Raman spectroscopy were loaded into a cryostat (THMS600, Linkam Scientific Instruments). The temperature in the cryostat was precisely controlled by an embedded heater in the system and liquid nitrogen from outsourcing for heating and cooling, respectively. The measurements of VO_2_ phases in a “VO_2_+NG” device, demonstrated in Fig. S5, were performed using a 50 × objective lens on a Renishaw micro-Raman/PL system equipped with an excitation laser (λ ~ 488 nm) which was focused on the area right next to the 3ω electrode (Fig. S2a). 190-μW laser power allowed acquiring Raman signals from the “VO_2_+NG” device within 0.5 s without any damage. The device was heated and cooled with maximum ramp rate limited by the cryostat system.

### Simulation of dynamic thermal switching of a TSS

Considering our “VO_2_+NG” device, we performed two dimensional time-dependent numerical calculation with a thin film structure of 100 nm Al heating layer, 95 nm Al_2_O_3_ layer, 200 nm VO_2_ actuation layer and 20 nm poly-Si layer, as described in the inset of Fig. S6. Its total length was 500 μm with temperature constant anchor regions at both ends (anchor length = 20 μm). Set on 273 K for an initial temperature of the environment and the whole structure, and the anchors used a fixed temperature of 273 K as the boundary condition. The temperature-dependent heat flux of the nanogap interface is derived from the calculated results (Fig. S8), while the heat fluxes of the other boundaries are 5 Wm^−2^ K^−1^) to imitate the air cooling in the ambient environment. The parameters for VO_2_ properties such as density^39^, heat capacity (including latent heat)^40^, Young’s modulus and Poisson’s ratio^41^, electrical conductivity and thermal conductivity are carefully chosen using literature and experimental or analytical values in this work. The material properties of Al, poly-Si and Al_2_O_3_ are based on build-in parameters in the COMSOL Multiphysics. Their thermal expansion is neglected, because thermal expansion is much weaker in the experiments compared to the transformaiton strain during the VO_2_ phase transition^37^. A stepwise voltage input was supplied to the Al layer, which heats up the entire system to ~343 K. It should be noted that the temperature of VO_2_ is evaluated at the geometrical central point of the VO_2_ layer.

### Data Availability

The datasets generated during and/or analysed during the current study are available from the corresponding author upon request.

## Electronic supplementary material

Supplementary information
