# Supplementary material for: Enhancing Modulation of Thermal Conduction in Vanadium Dioxide Thin Film by Nanostructured Nanogaps
Source: Sci Rep. 2017 Aug 2;7:7131. doi: 10.1038/s41598-017-07466-4 (PMC5540922; doi:10.1038/s41598-017-07466-4)
Supplement: Supplementary file 1 — Supplementary information [file 41598_2017_7466_MOESM1_ESM.doc]

Supplementary Information

Enhancing Modulation of Thermal Conduction in Vanadium Dioxide Thin Film by Nanostructured Nanogaps

Hwan Sung Choe, Joonki Suh, Changhyun Ko, Kaichen Dong, Sangwook Lee, Joonsuk Park, Yeonbae Lee, Kevin Wang, and Junqiao Wu*


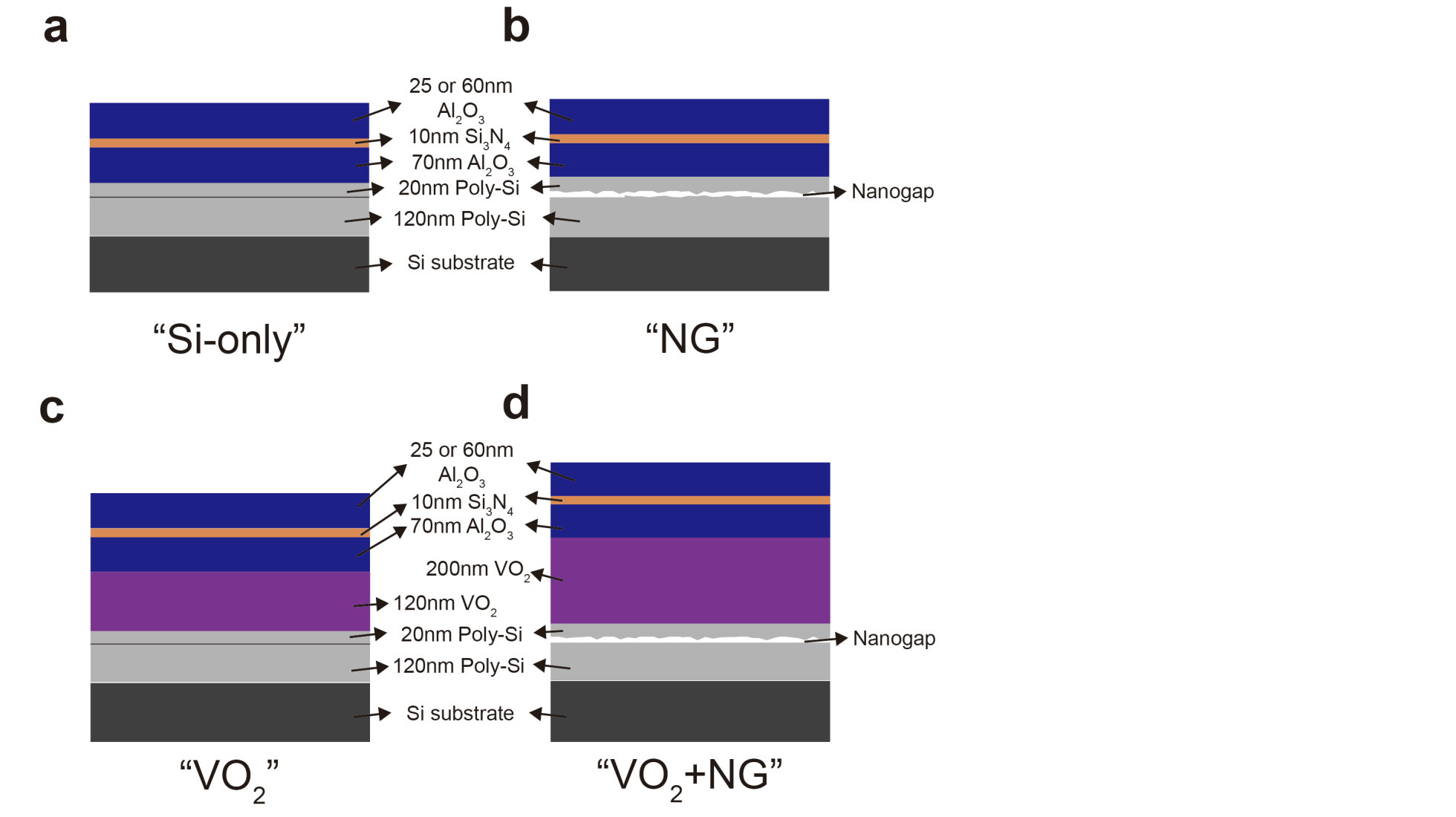


**Figure S1. Devices for differential 3ω measurements.** (a) Schematic of “Si-only” device. This device is used to extract thermal conductance of nanogap (NG), VO_2_, and VO_2_+NG by comparison of 3ω data with (b), (c), and (d) devices, respectively. (b-d) Schematics of “NG”, “VO_2_”, and “VO_2_+NG” devices. It is worth noting that all devices were fabricated with identical growth conditions and processes (Fig. S9) as the “VO_2_+NG” device except formations of (a) VO_2_+NG, (b) VO_2_, and (c) VO_2_ thickness+NG. The blue, purple, light grey, orange, red, and dark grey colors in schematics of (a-d) represent aluminium oxide, vanadium dioxide, polycrystalline silicon, silicon nitride, aluminium, and silicon substrate, respectively.


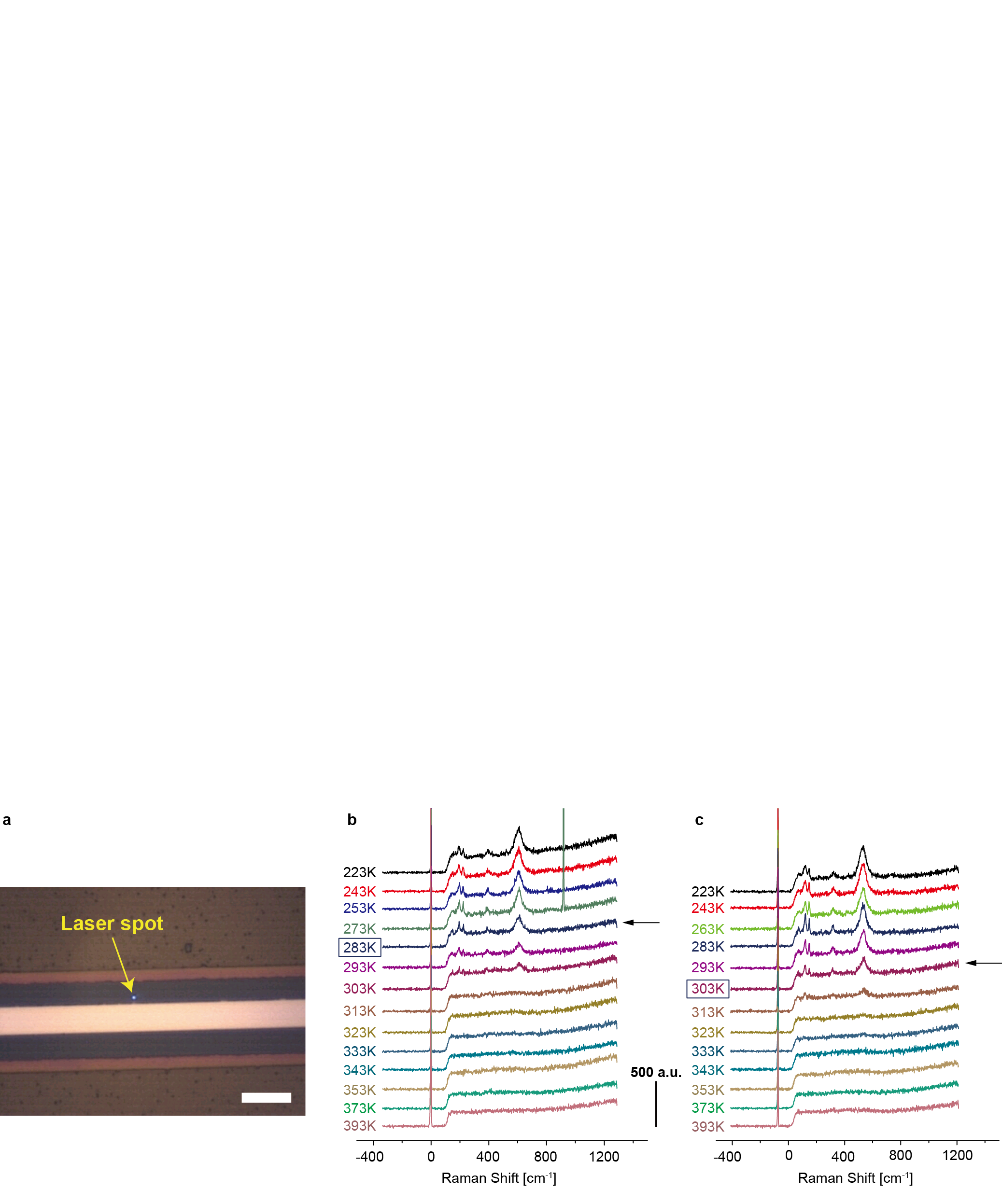


**Figure S2. Phase transitions of VO_2_ film in the thermal switching structure.** (a) Position of focused laser spot on thermal switch device for Raman spectroscopy. Based on the device structure (Fig. S9h**,** next to the top Al) and penetration depth of 488 nm argon-ion laser used for our Raman spectroscopy, the acquired Raman spectra at the position can represent the results of VO_2_ film in the TSS. Scale bar: 20 μm. (b-c) Raman spectra of (b) “VO_2_+NG” Device2 and (c) “VO_2_+NG” Device1 as increases of temperature, respectively. The black arrows point at the beginning temperature of the VO_2_ phase transition where the peak of 610 cm^-1^ begins to change drastically.


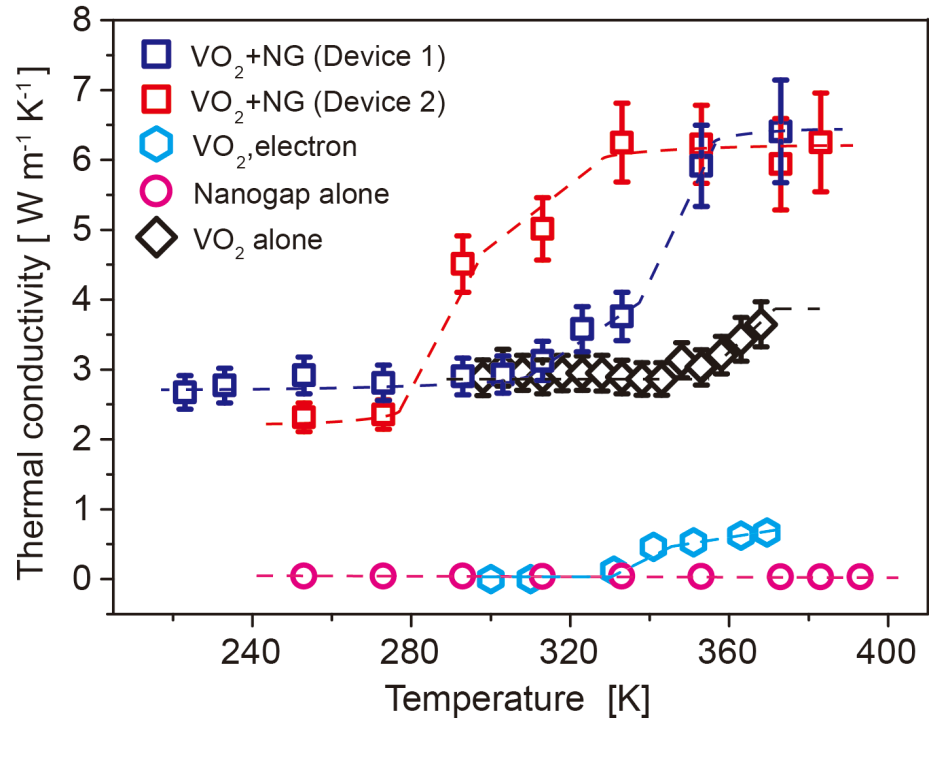


**Figure S3. Thermal conductivity of devices.** The effective thermal conductivity of the nanogap, VO_2_, “VO_2_+NG” Device1, and “VO_2_+NG” Device2 as a function of temperature. All results were obtained by the differential 3ω method with “Si-only” control devices (Fig. S1a), except the VO_2_ electronic thermal conductivity which was calculated by a combination of the Wiedemann-Franz law and of the measured electrical conductivity of the VO_2_ film. The dashed lines are given as guides to the eye.


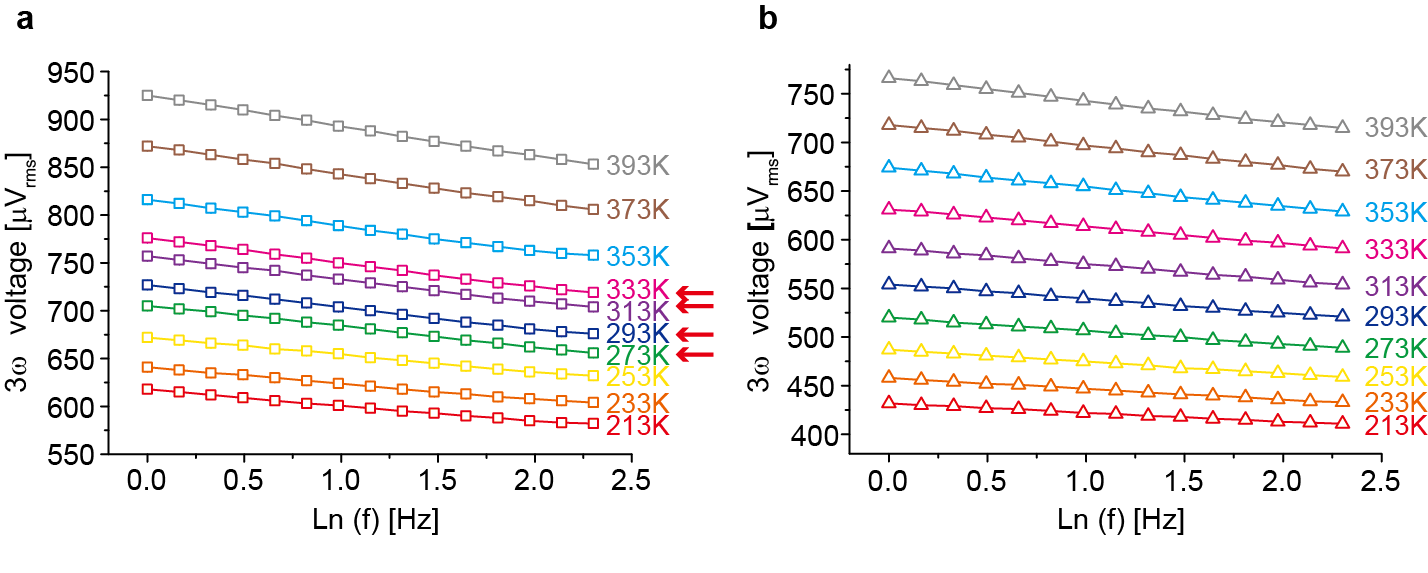


**Figure S4. 3ω signals of the thermal switching structure.** 3ω voltage of in-phase versus the first harmonic frequency of (a) the TSS (“VO_2_+NG” Device2), and (b) the control device (“Si-only”) as the global temperature increases. The red arrows point at the temperatures of the insulator-to-metal transition of VO_2_ in the TSS, which is confirmed by Raman spectroscopy (Fig. S2b).


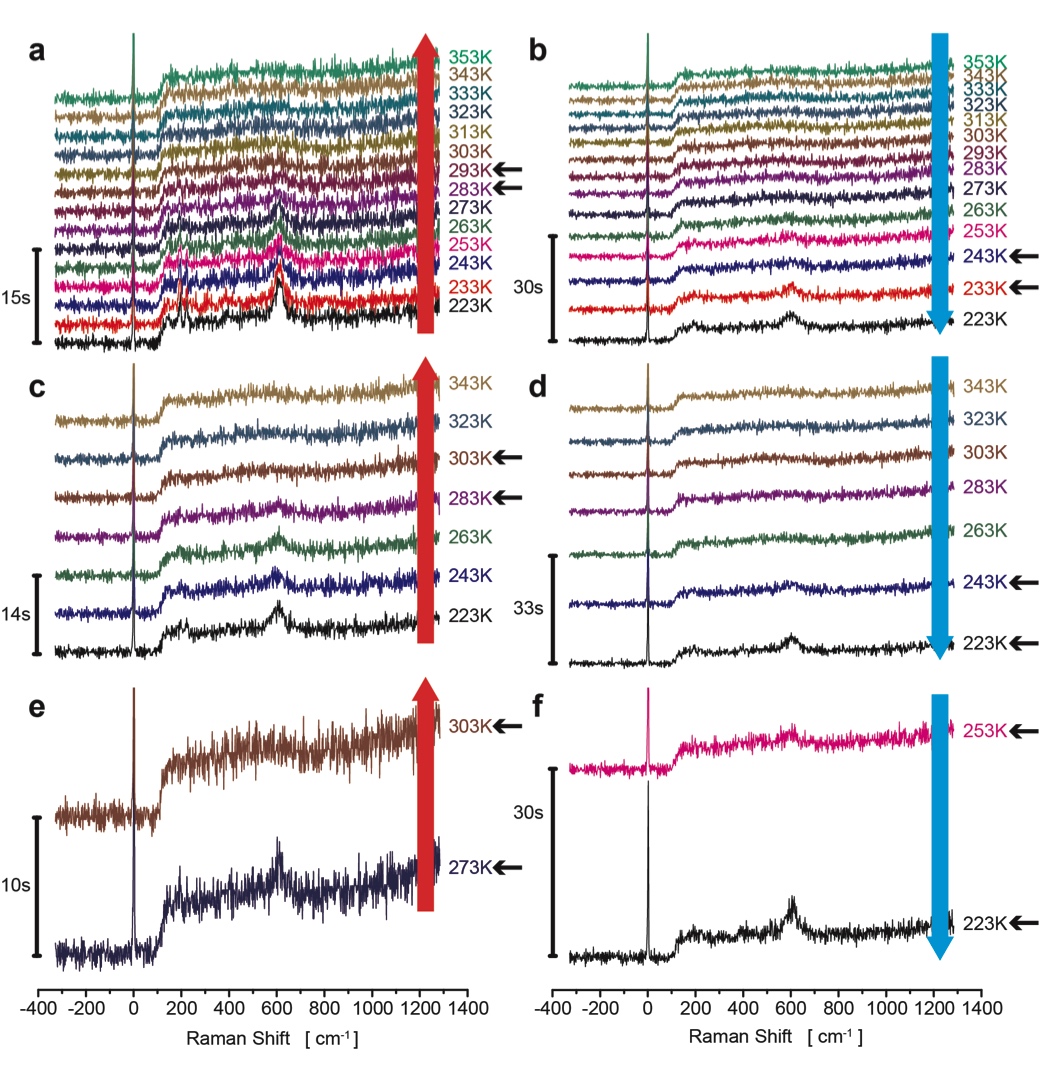


**Figure S5. Dynamic switching of VO_2_ phases in a TSS by changes of temperatures.** Raman spectra of VO_2_ layer in “VO_2_+NG” Device1 (Fig. S2a) as (a, c, and e) heating and (b, d, and f) cooling with (a-b) 10 K, (c-d) 20 K, and (e-f) 30 K changes of temperature between spectrum acquisitions, respectively. The y-axis represent the acquisition time to obtain one Raman spectrum to the other and the black arrows point at the temperature of evident VO_2_ phase transition on the basis of the change of the peak of 610 cm^-1^.


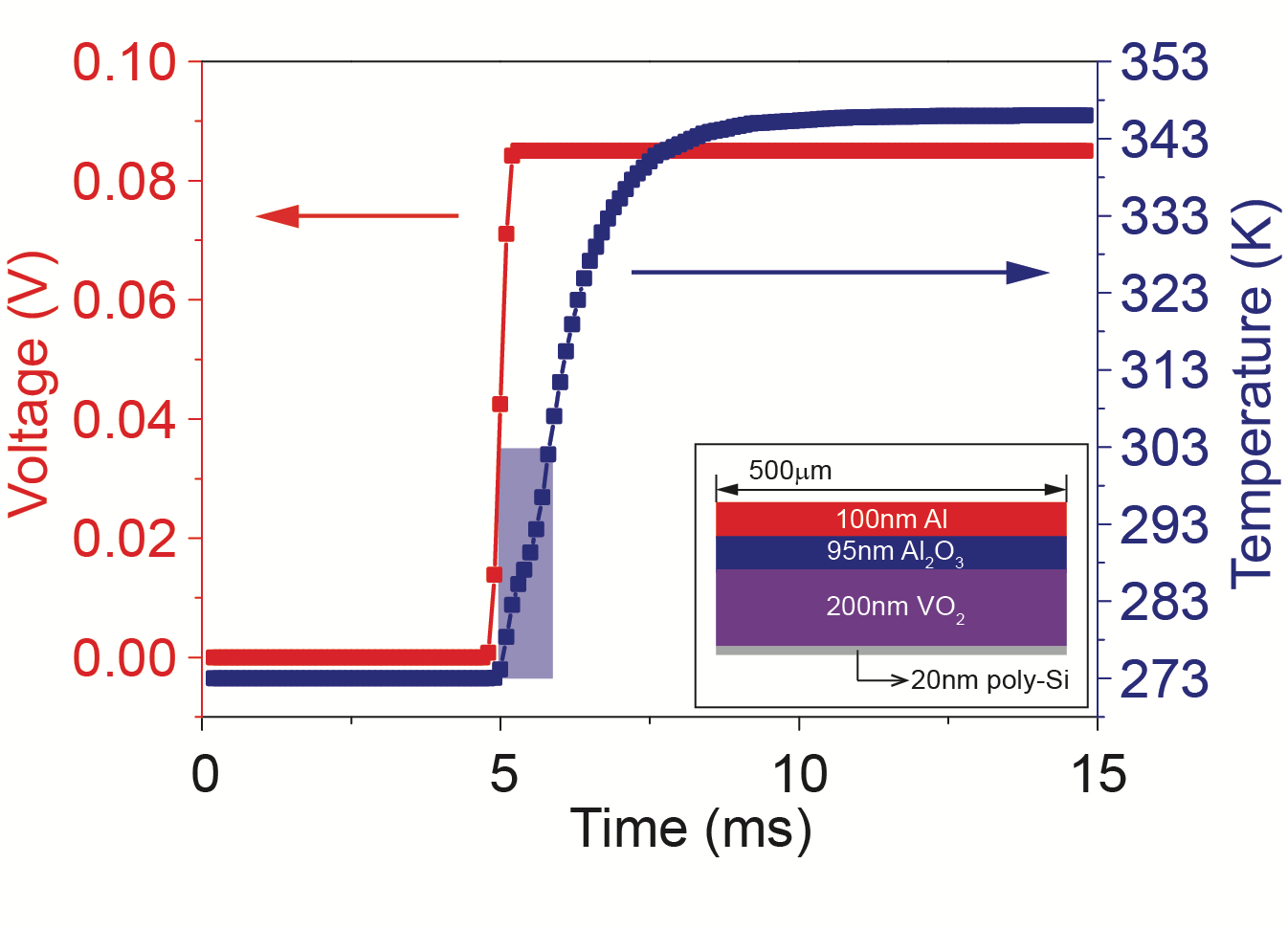


**Figure S6. Simulation of dynamic thermal switching of “VO_2_+NG” Device1 by Joule heating.** Temperature change at the center of 200 nm VO_2_ layer in the “VO_2_+NG” Device1 (blue curve) diven by the stepwise voltage input (red curve) in 100 nm Al as a function of time. Inset (not to scale) shows the dimensional information of each layer in the simulated “VO_2_+NG” Device1. Given the demonstrated temperature range for VO_2_ phase transition in the “VO_2_+NG” Device1 during heating (Fig. S5e), The maximum switching speed was estimated to be up to ~1 ms. The shaded rectangle in the graph corresponds to the temperature range over which the VO_2_ phase transition in the “VO_2_+NG” Device1 occurred.


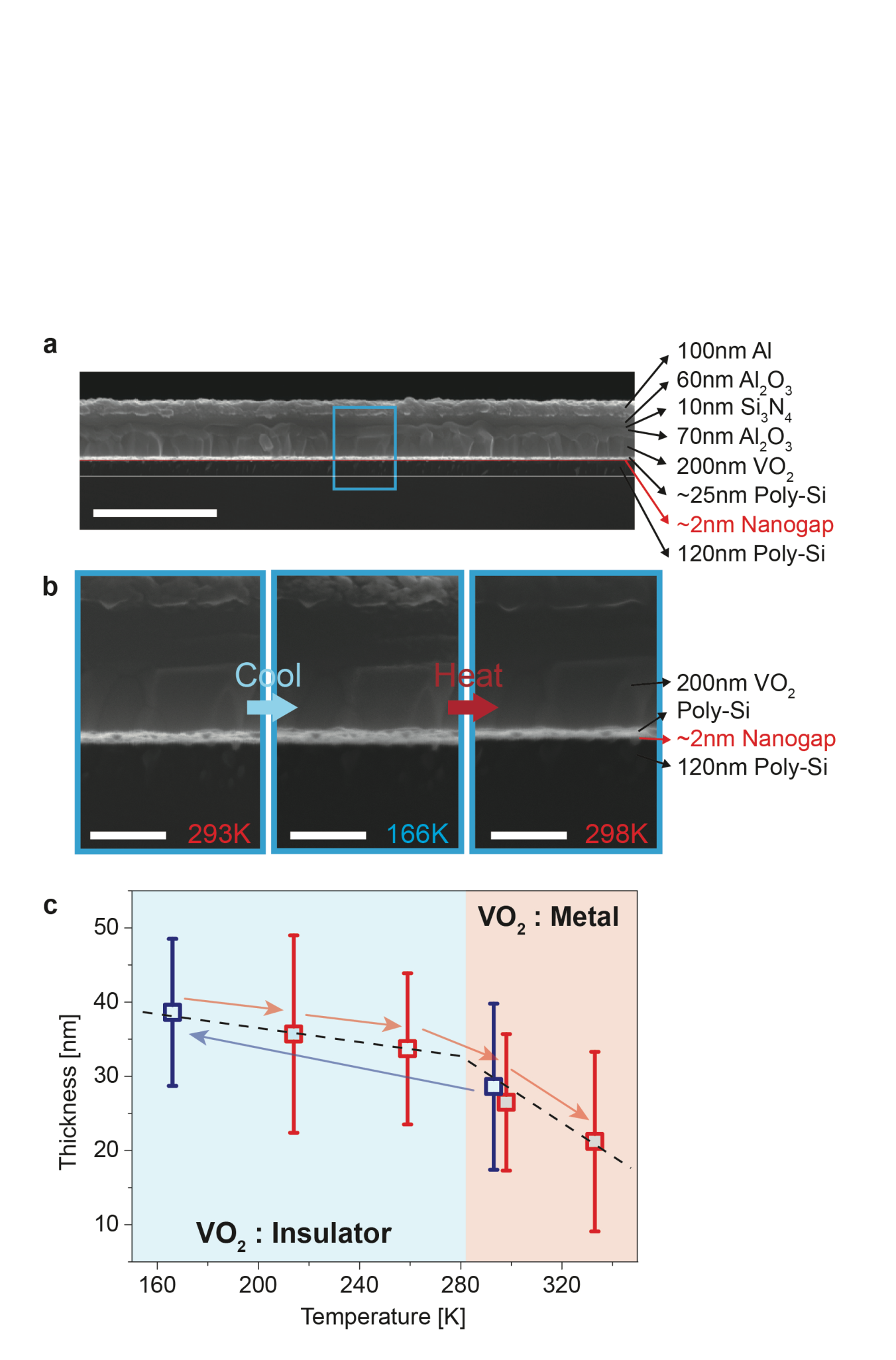


**Figure S7. Modulated nanogap by phase transition of the VO_2_ thin film in a thermal switching structure.** (a) A cross-sectional SEM image of the device at room temperature. The information of all layers was from TEM analysis (Fig. S9j) including the nanogap (red line). A cyan-colored rectangular marks the area taken during *in-situ* SEM (250,000× magnification) for monitoring poly-Si / nanogap / poly-Si / VO_2_ film stack at variable temperatures. Scale bar : 1 μm. (b) *In-situ* SEM images of the thermal switching layers during cooling and heating. It should be noted that high brightness of the poly-Si near the nanogap, compared to the bottom poly-Si, may arise from height difference caused by the preparation of the cross section. All images were taken after a 5 min wait for temperature stabilization and an e-beam focus on the poly-Si layer on the nanogap. Scale bars: 200 nm. (c) Apparent thickness of the poly-Si layer above the nanogap versus temperature during cooling and heating. The thicknesses at each temperature were measured from *in-situ* SEM images, and the error bars were evaluated from the roughness of top and bottom boundary of the poly-Si on the nanogap. The phase transition temperature of VO_2_ in the TSS (283 K) is obtained from the Raman spectroscopy (Fig. S2).


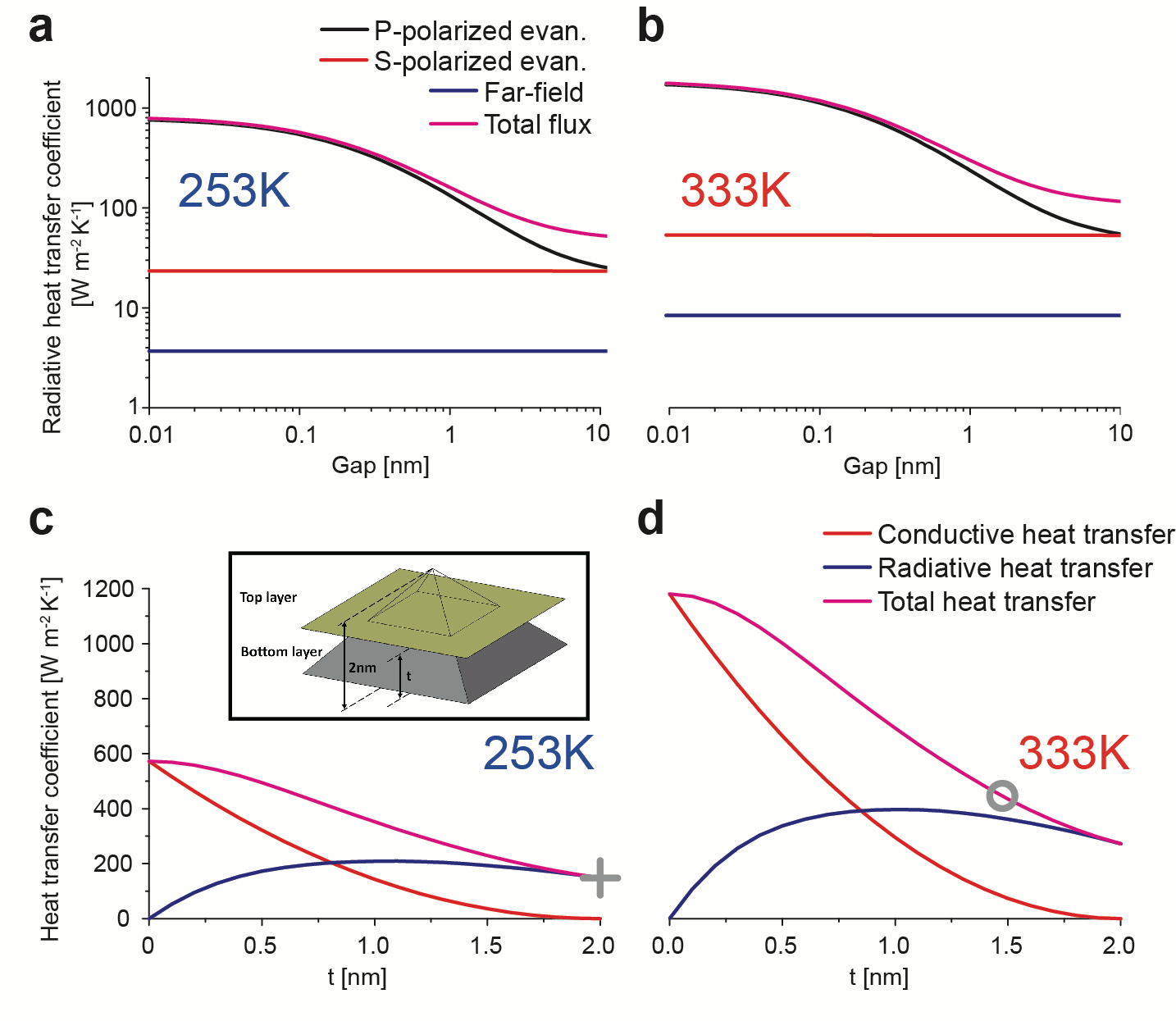


**Figure S8. Calculation of heat transfer coefficient across variable gaps.** (a-b) Radiative heat transfer coefficients across variable gaps between two parallel poly-Si planes at (a) 253 K and (b) 333 K. The calculations are performed with 1 K temperature difference between the planes. (c-d) Total heat transfer coefficients associated with radiative and conductive heat transfers as a function of gap size between a tetragonal pyramid and a plane of poly-Si (inset) at (c) 253 K and (d) 333 K. The temperature difference between the top plane layer and the bottom layer of the pyramid is set to 1 K. Obtained from the experimental switching performance of the TSS (Fig. 3), the grey cross and circle in the plot of (c) and (d) indicates estimated total heat transfer coefficients before and after the phase transition of VO_2_ film in the TSS, respectively.


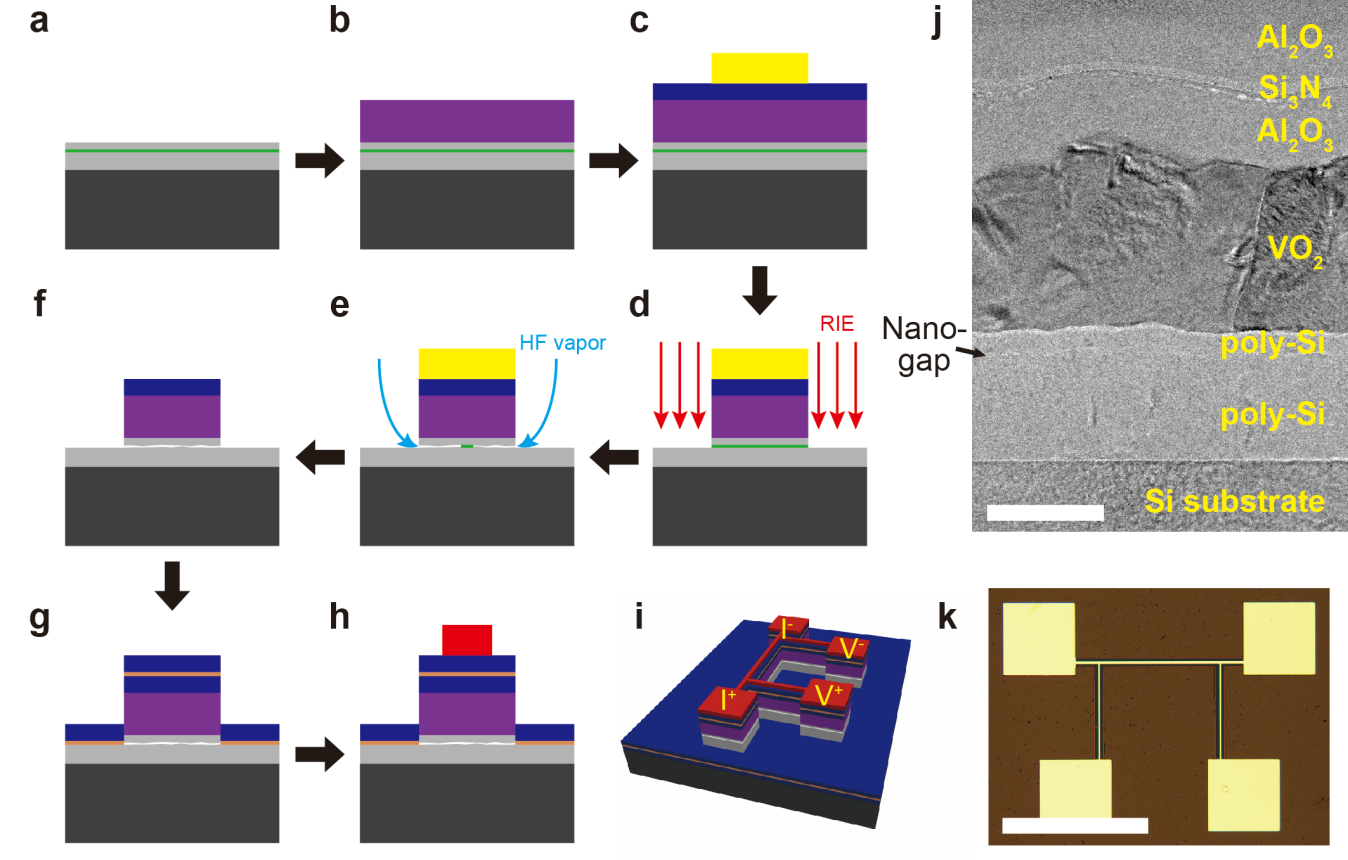


**Figure S9. Fabrication of thermal switching structure for 3ω measurement.** (a) Growth of 120 nm poly-Si/20 nm LTO/20 nm poly-Si thin-film stack on a Si substrate by low-pressure chemical vapour deposition. (b) Growth of 200 nm VO_2_ film by PLD. (c) Deposition of 70 nm Al_2_O_3_ by ALD and 70 nm Au by an e-beam evaporator with photolithography for a protection mask. (d) Etch of Al_2_O_3_/VO_2_/poly-Si/LTO layers with the Au-protection mask by RIE. (e) Selective removal of LTO layer without damages on VO_2_ thin film by HF vapour. (f) Removal of top Au-protection mask by iodine-based Au etchant. (g) Deposition of 10 nm/60 nm (or 25 nm) Si_3_N_4_/Al_2_O_3_ oxides by PECVD and ALD, respectively. (h) 1 nm/100 nm Cr/Al metallization of four-probe electrodes on the top of film stack by e-beam evaporation after photolithography. (i) 3D illustration of (h). The I^+^, I^-^, V^+^, and V^-^ indicate the connections for 3ω measurements. (j) Cross-sectional AC-HRTEM image of the typical thermal switch device, (h). This device has 60 nm Al_2_O_3_ for the topmost oxide. Scale bar: 100 nm. (k) Optical image of the typical thermal switching device for 3ω measurement, (i). Scale bar: 600 μm. The blue, purple, light grey, orange, red, dark grey, green, and yellow colors in schematics of (a-i) represent aluminium oxide, vanadium dioxide, polycrystalline silicon, silicon nitride, aluminium, silicon substrate, low temperature silicon dioxide, and gold, respectively.


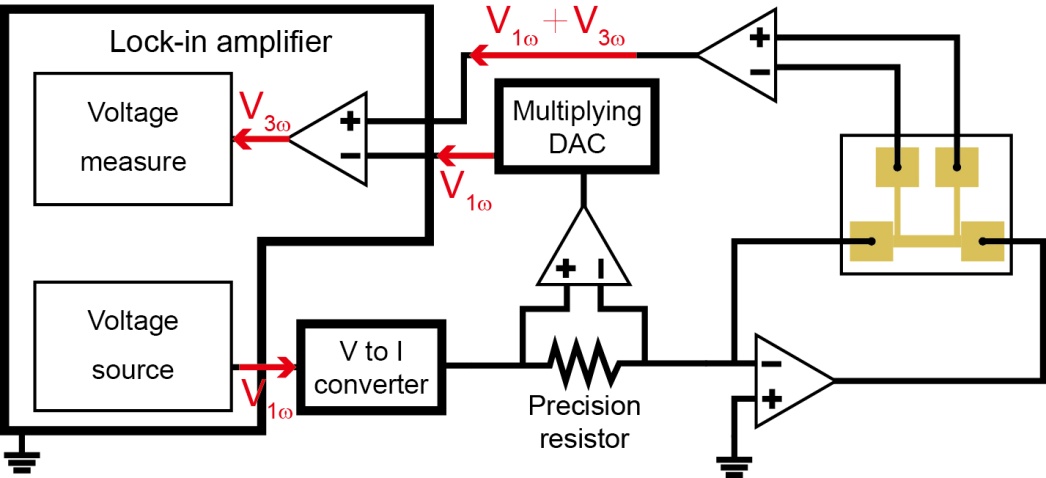


**Figure S10. Experimental setup for the 3ω method.** Schematic of the experimental setup for 3ω measurements. The first harmonic current generates the first and the third harmonic voltage in the Al heater. The high-precision resistor with multiplying DAC was used to cancel out the first harmonic voltage and to capture the low-noise third harmonic voltage in the lock-in amplifier.


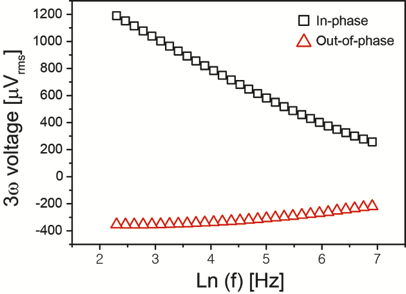


**Figure S11**. **Calibration of our home-made 3ω measurement system**. 3ω voltages of in-phase and out-of-phase were obtained from a fused silica substrate at room temperature. The thermal conductivity of the sample was revealed to 1.22 W m^-1^ K^-1^, calculated by slope method.^[1]^


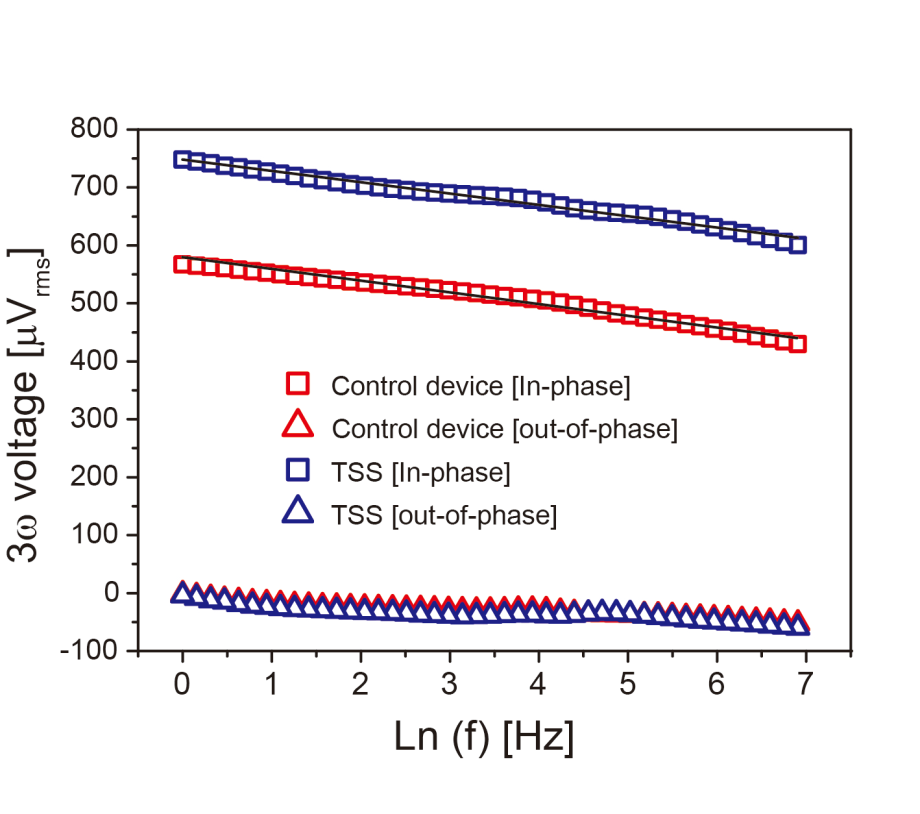


**Figure S12. Typical 3ω signals of the thermal switching device and control device.** 3ω voltage of in-phase and out-of-phase from the TSS and the control device, respectively, at 300 K as a function of the first harmonic frequency. The black lines show linear fitting of the in-phase data.

**Table S1. Comparison of out-of-plane thermal switching performances.**

|  | ON/OFF ratio | Switching speed | Switching range of thermal conductivity [W m^-1^ K^-1^] |
| --- | --- | --- | --- |
| Our TSS | ~ 2.7 | > 1 ms (simulated data) | 2.3 – 6.25 |
| VO_2_^[3]^ | ~ 1.6 | Not discussed | 3.5 – 5.5 |
| - GeSb_4_Te_7_^[4]^ | ~ 2 | Not discussed | 0.4 – 0.8 |
| Pb(Zr_0.3_Ti_0.7_)O_3_^[5]^ | 1.1 | < 300 ms  (experimental data) | 1.03 – 1.14 |

**Table S2. Thermal switching efficiency of the TSD compared to VO_2_ film alone.**

|  | Energy density needed for activation, E [J m^-2^] | Thermal switching ratio, (G_on_ – G_off_) / G_off_ | Thermal switching ratio, (G_on_ – G_off_) / G_off_ / E [m^2^ J^-1^] |
| --- | --- | --- | --- |
| VO_2_ alone | ~ 47^[1, 2]^ | 0.21 | 0.0047 |
| VO_2_ + NG (Device 2) | ~ 47^[1, 2]^ | 1.6 | 0.034 |

Solid-state thermal switches require external energy input to switch between the ON and OFF states. The energy input in our solid-state TSS is used to provide the latent heat for the VO_2_ phase transition. To gauge the merit in terms of energy efficiency of a thermal switch, we define the efficiency as

.

The thermal switching efficiency of “VO_2_” and “VO_2_+NG” Device2 at room temperature are listed in Table S2. ξ of “VO_2_+NG” Device2 is one order of magnitude larger than the “VO_2_” device due to the improved thermal switching ratio.

**Supplementary Note 1 | Typical 3ω voltage of thermal switching structure**

To measure in-phase and out-of-phase signals of the 3ω voltage across the metal line, a lock-in amplifier and differential amplifiers were employed. In particular, the multiplying digital-to-analog converter (DAC) with a high-precision resistor was added to cancel out the 1ω signal during the acquisition of the 3ω voltages (See Fig. S10). The frequency-dependent ΔT of the TSS and the control device calculated from in-phase 3ω voltages demonstrate a non-linear and a linear region in the 10-100 Hz and 1-10 Hz range, respectively (See Figure S12). Considering thermal penetration depth of the TSS, the 1-10 Hz frequency range is long enough to allow heat to go deep over the thickness of the device and the control.^[6]^ Hence, the linear region in 1-10 Hz was used for the differential 3ω method to obtain thermal conductivity and conductance of the devices. We also note that the slope in the ΔT ~ ln(ω) curve equals , and the calculated thermal conductivity of the substrate, κ_S_, from the devices are 100~125 W m^-1^ K^-1^, close to reported 300 K thermal conductivity of the substrate, single crystalline silicon.^[7]^

**Supplementary References**

1. C. N. Berglund, H. J. Guggenheim, *Phys. Rev.* **1969**, 185, 1022.
2. J. Cao, E. Ertekin, V. Srinivasan, W. Fan, S. Huang, H. Zheng, J. W. L. Yim, D. R. Khanal, D. F. Ogletree, J. C. Grossman & J. Wu, *Nat. Nanotechnol.* **2009**, 4, 732.
3. D.-W. Oh, C. Ko, S. Ramanathan, D. G. Cahill, *Appl. Phys. Lett.* **2010**, 96, 151906.
4. S. Shin, H. K. Kim, J. Song, D. J. Choi, H. H. Cho, *J. Appl. Phys.* **2010**, 107, 033518.
5. J. F. Ihlefeld, B. M. Foley, D. A. Scrymgeour, J. R. Michael, B. B. McKenzie, D. L. Medlin, M. Wallace, S. Trolier-McKinstry, P. E. Hopkins, *Nano Lett.* **2015**, 15, 1791.
6. C. Dames, *Annu. Rev. Heat Transfer* **2013**, 16, 7.
7. M. Asheghi, M. N. Touzelbaev, K. E. Goodson, Y. K. Leung, S. S. Wong, *J. Heat Transfer* **1998**, 120, 30.
